# Allowing Unlimited PFAS Manufacturing Contradicts the Core Intention of the European Union’s PFAS Restriction Proposal

**DOI:** 10.1021/acs.est.6c04920

**Published:** 2026-04-23

**Authors:** Anna J. Miller, Amanda Rensmo, Ian Cousins, Rainer Lohmann, Gretta Goldenman, Dorte Herzke, Xenia Trier, Zhanyun Wang, Martin Scheringer

**Affiliations:** † Institute for Biogeochemistry and Pollutant Dynamics, Department of Environmental Systems Science, ETH Zurich, 8092 Zurich, Switzerland; ‡ Department of Environmental Science, 7675 Stockholm University, 10691 Stockholm, Sweden; § Graduate School of Oceanography, 54083 University of Rhode Island, Narragansett, Rhode Island 02882, United States; ∥ Milieu Consulting SPRL, 1050 Brussels, Belgium; ⊥ Department of Environmental Chemistry, NILU, 9296 Tromsø, Norway; # University of Copenhagen, Department of Plant and Environmental Sciences (PLEN), Frederiksberg C, Thorvaldsensvej 40, 1871 Copenhagen, Denmark; 7 University of Southern Denmark, Department of Green Technology & Danish Institute for Advanced Study (DIAS), 5230 Odense, Denmark

**Keywords:** PFASs, hazardous properties, future emissions

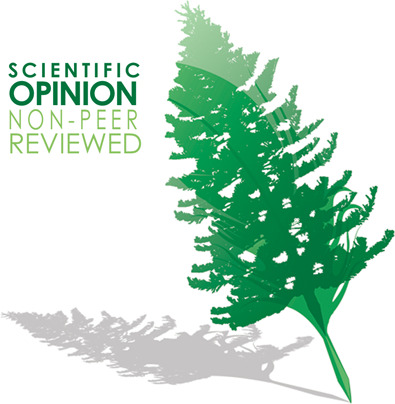

## Graphical abstract

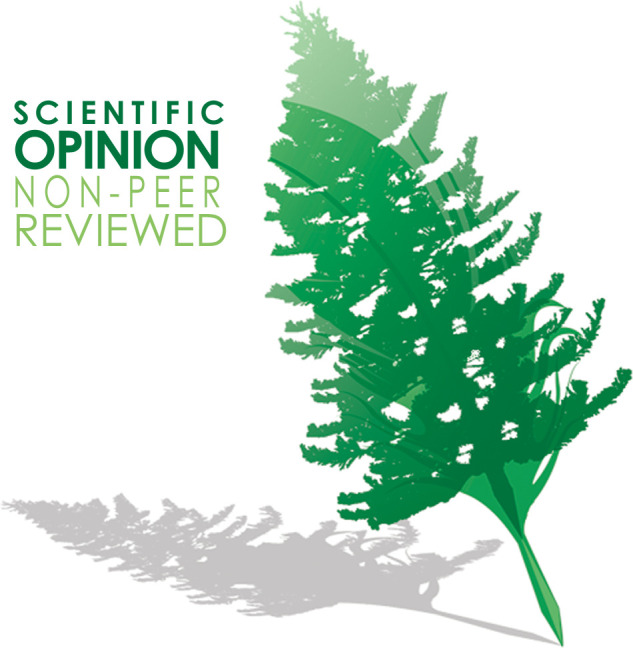

Emissions of per- and polyfluoroalkyl substances (PFASs) are a serious problem due to the extreme persistence of PFASs: once PFASs are present in the environment, it is technically and economically difficult, if not impossible, to remove them. Most PFASs have additional hazardous properties such as mobility, bioaccumulation, and (eco)­toxicity and/or are transformed in the environment into hazardous PFASs. The proposal for a universal PFAS restriction in the European Union, submitted by five European countries (dossier submitters) in 2023, addresses the risk of PFASs to human health and the environment by intending to restrict PFASs as a group.^1^ Its central ethos is clear: PFAS emissions must be minimized because no safe level of PFAS contamination exists.

The dossier submitters estimate future emissions of PFASs under “business-as-usual” conditions at ∼4.8 million t of PFASs in the European Economic Area (EEA) over a 30-year period, plus 50 000 t of emissions from PFAS manufacturing.^2^ In the dossier, the term “PFAS manufacturing” is used to refer only to the synthesis of PFASs, not the downstream processing of PFASs for use in goods and industrial processes; this terminology is adopted in the present Viewpoint. This distinction is significant because viable alternatives for most PFAS uses already exist or are in development,^3,4^ but for PFAS manufacturing, the idea of “alternatives” has no meaning, as PFASs are being made rather than used.

To accommodate uses of PFASs without suitable available alternatives today, the proposal allows for derogations. The updated 2025 version presents four restriction options (ROs) for PFAS manufacturing. RO1 is a full ban on manufacturing, use, and placing on the market of PFASs. RO2 allows manufacturing only to supply derogated uses for up to 12 years after the transition period. RO3a and RO3b would allow any PFAS manufacturing with emission control measures, either for 12 years (RO3a) or for an unlimited time (RO3b).

For the RO3 options, the dossier proposes average emission factors (AEFs) in percent, defined as the annual mass of PFASs emitted divided by the annual mass manufactured at a site (×100%). Separate AEFs are proposed for emissions from polymerization-aid technology in fluoropolymer manufacturing (0.0036% to all compartments from 2030 on) and for all other PFAS emissions from manufacturing sites (0.01% to all compartments from 2030 on). These factors are intended to represent the “best available techniques” (BATs) and to serve as the basis for emission control.

In the dossier, the need for the proposed universal PFAS restriction was justified in terms of the extensive health costs for the EEA and other societal burdens. However, in the socioeconomic assessments of individual sectors affected by the restriction, the overall societal burden was not taken into account. Costs were defined quite narrowly as economic impacts on companies, consumers, and employment, whereas benefits were defined solely as avoided emissions. For PFAS manufacturing, the dossier submitters concluded that all ROs have “high effectiveness” (high emission reduction), but that RO1, RO2, and RO3a have “high costs” due to business closures. RO3b, in contrast, was deemed as having only “moderate costs”, because continued manufacturing income would offset the costs of implementing emission control practices and there would be fewer business closures. RO3b was therefore presented as the preferred derogation.

In March 2026, the Risk Assessment Committee (RAC) and the Socioeconomic Assessment Committee (SEAC) of the European Chemicals Agency (ECHA) published their scientific opinions.^5,6^ RAC concluded that RO1 is the preferred risk-reduction option but stated that if use derogations are granted, RO3 (with emission controls) would be the most appropriate measure to reduce the risk. RAC also noted that additional risk management measures would be required and that they could not assess the technical feasibility of the proposed limits. SEAC found that RO3b may mitigate costs relative to other options because manufacturers would have a longer period to recover investments in emission controls.

Although RO3b is favored by both the dossier submitters and ECHA committees, we identify serious concerns that make RO3b incompatible with the proposal’s core intention of minimizing PFAS emissions.

## Continued PFAS Manufacturing Guarantees Continued PFAS Emissions

The central, and most risky, part of RO3b is its time-unlimited derogation for PFAS manufacturing. Without a time limit, there is no regulatory incentive for manufacturers to invest in innovation that enables a phase-out (demand for PFASs in the EU may decrease due to their use restrictions, but manufacturers may then increase exports). Even with emission controls, zero emissions cannot be achieved and, under RO3b, are not even intended to be achieved. Allowing manufacturing to continue indefinitely, therefore, ensures that PFAS emissions will also continue indefinitely, adding to an already excessive environmental burden.

Unlimited manufacturing volumes further amplify this problem. Continued synthesis feeds PFASs into downstream use and end-of-life stages, where additional emissions occur. In contrast, time-limited derogations such as RO2 and RO3a allow emissions to be halted at the source and eventually approach zero.

## The Proposed Average Emission Factors Are Not Emission Limits

The proposed AEFs are also problematic because they are factors, relative to the production volume, such that emissions grow with growing production volume. These AEFs are not absolute limits to PFAS emissions. There is no mechanism in the current proposal that would limit the total emissions, not even the total emissions per site, as emissions scale with production volumes.

The values of the proposed AEFs also raise concerns. The AEFs for “polymerization-aid technology” were derived from the Fluoropolymer Product Group’s “Responsible Manufacturing Project”,^7^ while the 0.01% AEF for all other PFAS emissions is based partly on the BAT for the destruction or recovery of trifluoromethane. If AEFs are based on BATs, the AEFs should be periodically updated to reflect technological improvements, yet no such mechanism is included.

Due to the unknown composition of emissions, measured emissions represent, in most cases, only a fraction of total PFAS emissions. Targeted analysis captures only a small subset of PFASs, and even nontarget methods cannot fully characterize the complex mixture of fluorinated organics emitted from manufacturing. Fugitive emissions are also difficult to quantify. Collectively, these limitations mean that relying solely on measured emissions to implement AEFs will likely underestimate true releases.

## Alternative Regulatory Approaches Are Needed

Given these concerns, RO3b should not be considered an appropriate measure for minimizing PFAS emissions. Allowing unlimited manufacturing volumes combined with emission controls with average and unchanging relative emission factors that are challenging to enforce will result in an indefinite amount of PFAS emissions over time in Europe and globally, gravely harming ecosystem and human health instead of protecting them. RO3a and RO2, by contrast, incorporate time limits that align with the proposal’s intention to phase out PFAS manufacturing and use.

We propose two alternative approaches consistent with the scientific evidence and the intention of the restriction proposal. First, PFAS manufacturing volumes should be strictly limited, e.g., through overall volume caps, to prevent any increase in manufacturing and associated emissions. Similar to the way chlorofluorocarbons were phased out under the Montreal Protocol, these volume caps could be based on the quantities required to supply essential (i.e., derogated) uses in the EEA, while the number of use-specific derogations should be kept to a minimum. If volume caps are considered unfeasible by the decision makers, a second option is to employ a time-limited derogation for PFAS manufacturing combined with fixed (not relative) emission limits. These limits should be stringent, process-specific, aligned with BAT, and easy to implement and enforce, with regular reevaluations as technologies improve. Without such measures, the universal PFAS restriction risks promoting the very emissions it seeks to prevent.

## ∇

Retired.
